# Cross-Cultural Adaptation and Psychometric Validation of the Arabic Chronic Venous Insufficiency Quality-of-Life Questionnaire (CIVIQ-20) in Saudi Arabia

**DOI:** 10.3390/jcm15197558

**Published:** 2026-09-29

**Authors:** May S. Alabdullateef, Rehab F. M. Gwada, Mohammed T. A. Omar

**Affiliations:** 1 Department of Physical Therapy, Prince Sultan Military Medical City, Riyadh 12233, Saudi Arabia; 2 Department of Rehabilitation Health Sciences, College of Applied Medical Sciences, King Saud University, Riyadh 12372, Saudi Arabia; rgwada@ksu.edu.sa

**Keywords:** chronic venous insufficiency, CIVIQ-20, Arabic, quality of life, reliability, validity, Saudi Arabia

## Abstract

**Background/Objectives:** Chronic venous disease significantly impairs health-related quality of life (HRQoL). Although the Chronic Venous Insufficiency Questionnaire (CIVIQ-20) is widely used internationally, a validated Arabic version is lacking. The objectives of this study were to translate and culturally adapt the CIVIQ-20 into Arabic and to examine its reliability, construct, and discriminative validity in Arab patients with chronic venous disease. **Methods:** Following standardized guidelines, the CIVIQ-20 was translated and cross-culturally adapted. For psychometric evaluation, 139 participants with chronic venous disease were selected. They completed the Arabic version of Chronic Venous Insufficiency Questionnaire-20 (CIVIQ-20-Ar) and 36-Item Short Form Health Survey (SF-36). Psychometric evaluation included internal consistency (Cronbach’s alpha) and test–retest reliability using the two-way random intraclass correlation coefficient ICC _(2,1)_. Minimal detectable change (MDC_95_) was assessed, along with both convergent validity (using the SF-36 as a comparator) and known-group validity (across CEAP severity stages), using seven predefined hypotheses. **Results:** The CIVIQ-20-Ar questionnaire had adequate internal consistency and very strong test–retest reliability. As hypothesized, the CIVIQ-20-Ar subscales and global index demonstrated a moderate-to-strong correlation with the SF-36 summary and domain scores (r = −0.42 to −0.79, *p* < 0.001), with the strongest correlation in the pain subscale (r = −0.79, *p* < 0.001). Known-group validity was established by significant score discrimination across advancing Clinical–Etiology–Anatomy–Pathophysiology (CEAP) severity stages (*p* < 0.01). **Conclusions:** The CIVIQ-20-Ar Questionnaire has adequate psychometric properties. Therefore, vascular surgeons, phlebologists, and rehabilitation specialists can use the CIVIQ-20-Ar in routine clinical practice and research to evaluate health-related quality of life in the Arabic-speaking population with chronic venous disease.

## 1. Introduction

Chronic venous disease (CVD) is a widespread vascular condition affecting millions worldwide, with a global prevalence ranging from 20% to 60% across adult populations. CVD encompasses a broad spectrum of clinical manifestations classified by the Clinical–Etiology–Anatomy–Pathophysiology (CEAP) framework, ranging from early mild features such as telangiectasias and varicose veins (C1–C2) to advanced functional impairments. This wide variation is largely attributed to inconsistencies in study populations, diagnostic criteria, imaging techniques, and risk factor exposures across regions [1,2,3]. For instance, a prospective international study investigating chronic venous disorders (CVDs) across Europe and the Middle East found that 63.9% to 83.6% of subjects met the criteria for CVD (CEAP stages C1 to C6) [4]. The term chronic venous insufficiency (CVI) is specifically reserved for advanced disease stages (C3–C6), where persistent venous hypertension leads to objective clinical sequelae such as lower-limb edema, skin trophic changes, stasis dermatitis, and active or healed venous leg ulcers. In the Middle East, epidemiological data remain limited; an early study across primary healthcare centers in three Saudi Arabian cities reported a venous disease prevalence of 45.6% [5]. Updated, large-scale studies are essential to establish accurate, contemporary national prevalence rates. These high prevalence and diagnostic variability highlight a major public health challenge that imposes a heavy physical, emotional, and economic burden on patients and healthcare systems alike [6,7].

The clinical presentation of CVD includes a broad spectrum of subjective symptoms and objective signs. Patients frequently report leg heaviness, pain, nocturnal cramps, and itching. Early clinical signs include pitting lower-limb edema and telangiectasias [1,2,3]. In advanced stages, trophic skin changes become evident, including skin hyperpigmentation and, ultimately, leg ulcers [1,2,3]. Beyond these primary clinical manifestations, the progressive nature of CVD significantly compromises physical function, emotional well-being, and overall health-related quality of life (HRQoL), with functional limitations worsening as patients progress from early disease to severe CVI [8].

Evaluating HRQoL through patient-reported outcome measures (PROMs) has become essential in modern clinical practice, and comparative effectiveness research is needed to monitor disease progression, risk-stratify patients, guide specialist referrals, and determine the efficacy of medical and rehabilitative interventions on overall well-being [6,9]. Given the chronic nature of CVI, treatment strategies increasingly focus on improving long-term health status and overall HRQoL [9,10,11]. While objective clinical measures such as the Clinical, Etiological, Anatomical, and Pathophysiological (CEAP) classification offer a robust standardized clinical tool for diagnosis, grading, and guiding targeted treatment regimens [10], they must be complemented by PROMs to capture each patient’s subjective functional and symptomatic experiences [9,10,11].

For these purposes, generic PROMs such as the Sickness Impact Profile (SIP), the Nottingham Health Profile (NHP), and the 36-item Short Form Health Survey (SF-36) are typically used to evaluate overall patient health. In addition, disease-specific PROMs designed explicitly for CVI offer significantly greater sensitivity and specificity to venous-related changes [10,11,12,13]. Disease-specific scales can identify the distinct benefits of specific treatments for venous insufficiency. Therefore, it is crucial to develop and culturally adapt specific HRQoL instruments for venous insufficiency that account for the patient’s perspective and provide attending physicians with fine-grained measurements of the disorder’s functional and psychosocial consequences [13].

The Chronic Venous Insufficiency Questionnaire-20 (CIVIQ-20) is the first quality of life (QoL) questionnaire specific to chronic venous disorders and is widely recognized for evaluating the impact of CVI on daily life. This 20-item questionnaire provides a global index and a profile across four QoL dimensions: psychological (nine items), pain (four items), physical (four items), and social (three items) [14,15,16,17]. It was initially developed in French [14], subsequently translated into English [16], and has been successfully validated across various languages, including Spanish [15], Dutch [18], Croatian [19], Turkish [20], and Greek [21]. However, its clinical utility in diverse global settings necessitates rigorous cross-cultural adaptation and psychometric validation to ensure that translated versions maintain both conceptual equivalence and linguistic relevance for the target population [22].

To date, the lack of a validated Modern Standard Arabic (MSA) version of the CIVIQ-20 restricts its use among Arabic-speaking populations, often forcing clinical practice to rely on informal translations or the English proficiency of attending physicians. This reliance compromises both diagnostic objectivity and cross-study comparability [22,23]. Although regional dialects are spoken in daily life by over 450 million people, MSA remains the universal register for formal communication, education, and healthcare documentation across the region [24]. Validating a culturally adapted MSA version is necessary. This validation can ensure broad accessibility across diverse populations and provide clinicians with a reliable tool for accurately monitoring patient outcomes and addressing the disease’s significant physical, emotional, and socioeconomic burdens [24]. Therefore, this study aimed to cross-culturally adapt the CIVIQ-20 into Arabic and establish its psychometric properties, focusing on internal consistency, stability over time, measurement error, and both construct and discriminative validity among Arabic-speaking patients with chronic venous insufficiency.

## 2. Materials and Methods

### 2.1. Study Design

This study was conducted in two distinct phases: Phase 1 focused on the cross-cultural adaptation and linguistic translation of the CIVIQ-20 into Modern Standard Arabic, and Phase 2 used a cross-sectional design to evaluate its validity and internal consistency with a test–retest subcomponent.

### 2.2. Phase 1: Translation and Cross-Cultural Adaptation

After obtaining formal permission from the original developer to translate and validate the instrument, the cross-cultural adaptation process was conducted based on the guidelines proposed by Beaton et al. [21]. This structured process included five steps: forward translation, synthesis, backward translation, expert committee review, and pre-testing to establish the Arabic version of the CIVIQ-20. First, an English version of the CIVIQ-20 was independently forward-translated into Modern Standard Arabic by two native Arabic-speaking bilingual translators (T1: a senior academic physical therapist specializing in vascular rehabilitation, and T2: a layperson translator with no medical background). Second, the research team and forward translators synthesized T1 and T2 into a single consensus Arabic version (T12). Third, two independent native English-speaking bilingual experts with no medical background were completely blinded. This was conducted to check whether the translated version reflected the same content as the original, producing two back-translation reports (BT1 and BT2). Fourth, an expert committee comprising methodologists (*n* = 2), healthcare professionals (*n* = 3), and translators (*n* = 4) reviewed all reports to establish semantic, idiomatic, experiential, and conceptual equivalence, producing the pre-final Arabic version. Fifth, to evaluate cognitive debriefing and item comprehensibility, this pre-final version underwent pilot testing with a purposive sample of 20 participants across various CEAP clinical stages. Semi-structured, face-to-face cognitive debriefing interviews were conducted using a “think-aloud” protocol. Participants were asked to complete the questionnaire, rate item clarity on a 4-point ordinal scale (1 = unclear to 4 = very clear), and paraphrase each item in their own words to verify that the translated text accurately captured the intended construct. To ensure that Modern Standard Arabic was universally accessible across varying literacy and educational levels without regional dialectal bias, the pilot sample intentionally included individuals with diverse educational backgrounds, ranging from basic secondary education to advanced university degrees. Participant feedback was reviewed by the expert committee to finalize the Arabic version of Chronic Venous Insufficiency Questionnaire-20 (CIVIQ-20-Ar) prior to psychometric testing.

### 2.3. Phase 2: Psychometric Validation of the CIVIQ-20-Ar

Participants were recruited through convenience sampling from the outpatient vascular departments at Prince Sultan Military Medical City and King Khalid University Hospital in Riyadh, Saudi Arabia, between October and December 2023. To be eligible for inclusion, participants had to be aged 18 years or older with a formal diagnosis of CVI established by a vascular consultant based on clinical presentation and physical examination. Clinical severity and disease stage were classified according to the CEAP standard [13], with eligible participants ranging from CEAP stages C1 through C6. Participants had to complete self-reported questionnaires independently and provide written informed consent prior to enrollment. Participants were excluded if they met any of the following criteria: CEAP stage C0, significant comorbidities that could independently confound pain or physical activity assessments, acute medical conditions, severe systemic illnesses (e.g., advanced cardiac or respiratory disease), or psychiatric or cognitive impairments that could impede accurate data collection or protocol compliance.

The study was designed and conducted in line with the Declaration of Helsinki [25]. Approval was issued by the Institutional Review Boards of the College of Medicine at King Saud University (Ref. No. E-23-7572) and Prince Sultan Military Medical City (Ref. No. E-2098). All participants provided written informed consent prior to inclusion in the study. The literature review and COSMIN guidelines revealed that a sample size of 100 participants is adequate for a valid study. This sample size was increased by 20% to compensate for incomplete and invalid data [26].

### 2.4. Outcome Measures and Procedure

Eligible participants underwent an initial interview and completed a survey about their demographic and medical history, which was confirmed with their medical records. In this session, participants filled out the Arabic versions of the CIVIQ-20 and SF-36. At the second visit, 60 participants completed the CIVIQ-20 within a one-week interval to assess test–retest reliability and ensure clinical stability, minimizing the risk of recall bias [15]. Participants completed the questionnaires independently.

#### 2.4.1. The CVIQ-20-Ar

The CIVIQ-20 is a disease-specific, self-administered outcome measure designed to evaluate HRQoL in participants with chronic venous disorders over a 4-week recall period [14,15,16,17]. The instrument assesses four different functional dimensions: pain (4 items: Q1–Q4), physical functioning (4 items: Q5–Q7, Q9), social functioning (3 items: Q8, Q10, Q11), and psychological well-being (9 items: Q12–Q20) [14,15,16,17]. Each item is rated on an equally weighted 5-point Likert scale ranging from 1 (very low impact) to 5 (very high impact), yielding an unadjusted overall raw score ranging from 20 to 100 [14,15,16,17]. Domain-specific raw scores are computed by summing their respective item scores, while the overall raw score aggregates all 20 items [14,15,16,17]. To enable standardized comparisons across domains containing unequal item counts, raw domain and global scores were linearly transformed into a standardized 0–100 index using the established formula [14,15,16,17]:

Standardized Score = [(S − m)/(M − m)] × 100

where “S” represents the observed final sum of item scores for a domain or global scale; m represents the minimum theoretical raw score; and M depicts the maximum theoretical raw score [14,15,16,17]. Under this linear transformation, global index scores range from 0 to 100, where higher scores represent lower HRQoL [14,15,16,17].

#### 2.4.2. The Arabic Version of the Short-Form Health Survey (SF-36)

The SF-36 is a widely recognized generic instrument used to assess HRQoL. It comprises 36 items across eight subscales that aggregate into two primary summary components: The Physical Component Summary (PCS) and the Mental Component Summary (MCS). The PCS encompasses physical functioning (PF; 10 items), physical role (RP; 4 items), bodily pain (BP; 2 items), and general health (GH; 5 items). The MCS comprises vitality (VT; 4 items), social functioning (SF; 2 items), emotional role (RE; 3 items), and mental health (MH; 5 items) [27]. Scores for each subscale range from 0 to 100, where 0 represents the poorest quality of life, and 100 indicates optimal health status using the standard SF-36 scoring algorithms [27]. The Arabic version of the SF-36 demonstrated satisfactory psychometric results [28].

### 2.5. Statistical Analysis

All statistical analyzes were conducted using International Business Machines Statistical Package for the Social Sciences (IBM SPSS) Statistics for Windows, Version 27 (IBM Corp., Armonk, NY, USA). The normality of continuous data distributions was formally assessed using the Shapiro–Wilk test. A *p*-value < 0.05 was established as the threshold for statistical significance. Sociodemographic characteristics, clinical variables, and HRQoL scores obtained from the CIVIQ-20-Ar and SF-36 were evaluated using descriptive statistics. Continuous variables were expressed as means and standard deviations (SDs), whereas categorical variables were presented as absolute frequencies and percentages (%).

#### 2.5.1. Reliability and Measurement Error

The internal consistency and reliability of the CIVIQ-20-Ar total scale and subscales were determined using Cronbach’s alpha (α). A Cronbach’s alpha of 0.70 or higher represents acceptable internal consistency [29].

To evaluate test–retest reliability, a subsample of stable outpatients (*n* = 60) was reassessed within a 3- to 7-day interval following initial administration. This short timeframe was specifically chosen because chronic venous disease (CVD) is a slowly progressive condition, making spontaneous clinical changes unlikely within one week while minimizing memory recall effects [30,31]. Furthermore, clinical stability during this interval was formally verified using the Global Rating of Change (GROC) scale administered before the retest session. Only participants who met the predefined stability criterion (GROC score between −2 and +2; *n* = 60) were included in the reliability analysis. This interval minimized recall bias while ensuring patients’ disease status remained stable [30,31].

Test–retest reliability was evaluated using the intraclass correlation coefficient, with respective 95% confidence intervals (CIs). The ICC was calculated based on a single measurement, absolute agreement, and two-way mixed effects (ICC2,1). An ICC higher than 0.90 represented excellent reliability; an ICC between 0.75 and 0.90 represented good reliability; an ICC between 0.40 and 0.74 represented moderate reliability; and an ICC lower than 0.4 represented poor reliability [30,31,32].

The measurement error and Standard Error of Measurement (SEM) were calculated using the following formula: SEM = SD × √ (1–ICC). Here, SD represents the sample’s baseline pooled standard deviation, and ICC is calculated (2,1) from the test–retest analysis [32,33,34]. Subsequently, the minimal detectable change at a 95% CI (MDC95) was computed to establish the smallest clinical change detectable beyond a measurement error [33,35]. The MDC (95) was calculated using the following equation: MDC (95) = SEM × 1.96 √2.

#### 2.5.2. Validity

Content validity was evaluated by nine experts (two vascular surgeons, five physiotherapists, and two specialized nurses). They were formally invited by email to participate in this study. They were asked to rate each item of the CIVIQ-20-Ar with a four-point Likert scale (4 = highly relevant/clear; 3 = quite relevant/ clear; 2 = somewhat relevant/clear; and 1 = not relevant/clear). The item content validity index (I-CVI) and scale-level CVI/average agreement (S-CVI/Ave) were calculated. The I-CVI is the proportion of content experts who gave an item a relevance rating of 3 or 4, while the CVI/Ave is calculated by dividing the sum of the I-CVIs by the total number of items. A scale with an I-CVI of 0.78 or higher and an S-CVI/Ave of 0.90 or higher was acceptable [35,36]. Floor and ceiling effects were assessed by determining the proportion of patients with the lowest and highest scale scores. Floor and ceiling effects below 15% are acceptable [30].

To assess the convergent validity of the Arabic CIVIQ-20-Ar, we formulated six predefined hypotheses examining the relationships between its domain and total scores and the corresponding subscales and summary components of the Arabic SF-36. The correlation values are interpreted as weak (0.21–0.39), moderate (0.4–0.60), strong (0.61–0.80), or very strong (0.81–1.0) [37]. We formulated and tested the hypotheses regarding the global index scores of the CIVIQ-20-Ar scale and its four domains: pain, physical functions, social functioning, and psychological well-being as follows:

**Hypothesis 1.**  *The physical domain of the CIVIQ-20-Ar would have a negative correlation (r = −0.40, p < 0.05) with the SF-36′s physical functioning subscale.*

**Hypothesis 2.**  *The pain domain of the CIVIQ-20-Ar would have a negative correlation (r = −0.40, p < 0.05) with the SF-36′s bodily pain subscale.*

**Hypothesis 3.**  *The psychological domain of the CIVIQ-20-Ar would have a negative correlation (r = −0.40, p < 0.05) with the SF-36′s emotional role subscale.*

**Hypothesis 4.**  *The social domain of the CIVIQ-20-Ar would have at least a negative correlation (r = −0.40, p < 0.05) with the SF-36′s social functioning subscale.*

**Hypothesis 5.**  *The total score of the CIVIQ-20-Ar would have a significant negative correlation (r = −0.40, p < 0.05) with the SF-36′s physical component summary (PCS).*

**Hypothesis 6.**  *The total score of the CIVIQ-20-Ar would have a significant negative correlation (r = −0.40, p < 0.05) with the SF-36′s mental component summary (MCS).*

The known-group validity of the Arabic CIVIQ-20 was evaluated by testing predefined hypotheses regarding expected differences in the global index score and subscale scores across clinical subgroups. It was hypothesized that patients with a more advanced stage of CVD would report significantly lower HRQoL compared to those in earlier stages. To ensure adequate statistical power and comparable group sizes, clinical stages were merged based on the CEAP classification into three distinct groups: C1 and C2; C3 and C4; and C5 and C6. Differences between these CEAP categories were evaluated using the Kruskal–Wallis test. Post hoc pairwise comparisons were applied to identify specific group differences. Overall, we formulated seven hypotheses to assess convergent and known-group validity. If more than 75% of the hypotheses were confirmed, the validity of the CIVIQ-20-Ar version would be sufficiently supported [38].

## 3. Results

### 3.1. Translation and Cross-Cultural Adaptation of the CIVIQ-20 into Arabic

The translation and cross-cultural adaptation process proceeded smoothly, yielding high linguistic and conceptual equivalence. During the expert committee review, minor adjustments were made to refine frequency descriptors and cultural relevance: in item 3, “fairly often” was adapted to “quite often” to better reflect the intended symptom frequency in Arabic. In item 10, the original phrase “going to parties” (with specific examples of “discos” and “cocktails”) was culturally adapted to “going to a social event”. This adaptation preserved the core construct of evaluating limitations to social life while ensuring appropriateness within Saudi socio-cultural contexts.

In the cognitive debriefing pilot phase (*n* = 20), participants across all educational levels (high school/less: *n* = 6; bachelor’s degree: *n* = 12; postgraduate: *n* = 2) completed the CIVIQ-20-Ar in an average of 5–8 min. All 20 participants confirmed that the questions were clear, unambiguous, and directly relevant to their venous disease symptoms. Paraphrasing during cognitive interviews verified that participants correctly interpreted the 4-week recall period and five-point Likert response scale. No participant reported difficulty understanding the Modern Standard Arabic text, confirming its broad comprehensibility regardless of educational attainment. The final version was formally reviewed and approved by the original developer (Supplementary File S1).

### 3.2. Psychometric Properties of the Arabic CIVIQ-20

#### 3.2.1. Participants’ Characteristics

**Figure 1:**
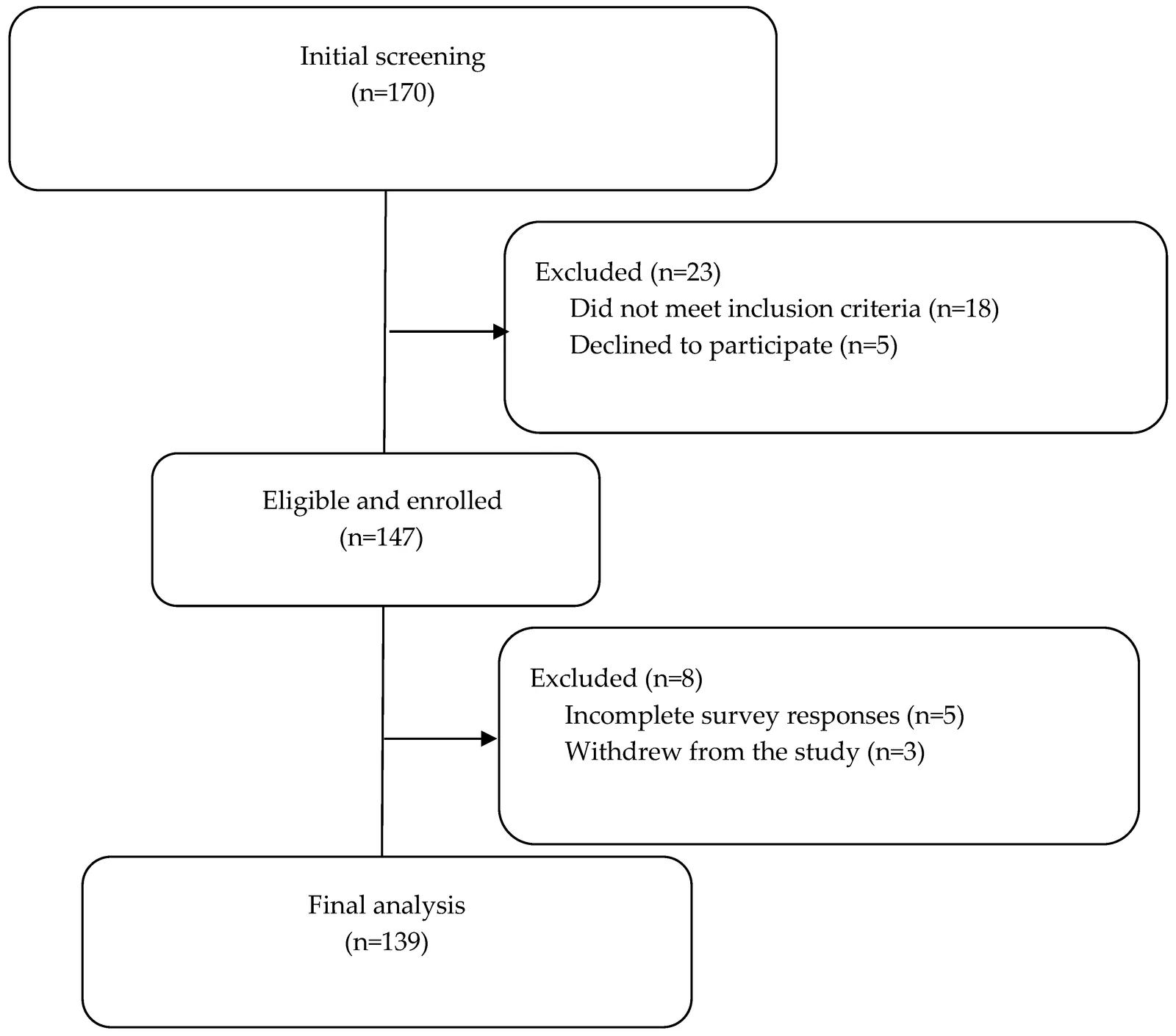
A flowchart detailing the enrollment of study participants (*N* = 139).

In the validation study (Phase 2), 170 participants were initially screened for eligibility. Following this, 23 participants were excluded: eighteen did not meet the specific inclusion criteria, and five declined to participate. Although 147 participants met the baseline eligibility requirements, an additional eight participants were excluded prior to data analysis because of incomplete survey responses (*n* = 5) or formal withdrawal from the study (*n* = 3). The final statistical analysis was conducted on a sample of 139 participants who provided complete datasets for the psychometric evaluation of the CIVIQ-20-Ar (Figure 1).

Table 1 provides details on the sample characteristics. The participants had a mean age of 47.13 ± 11.93 years. Most participants were female (84.17%) and married (86.33%). Clinically, most participants were categorized in the early clinical stages (C1–C2, *n* = 89, 64.0%). Moderate clinical presentation (C3–C4) was observed in 22.00% (*n* = 31) of participants, while the severe stage (C5–C6) accounted for 14.00% (*n* = 19). The HRQoL assessment had a mean CIVIQ-20-Ar global index score of 68.53 ± 19.96, with the highest level of impairment observed in the physical domain (37.94 ± 24.96) and the relative preservation of psychological well-being (25.43 ± 19.45). Generic SF-36 summary measures revealed component scores of PCS: 62.98 ± 25.29 and MCS: 64.73 ± 20.26.

#### 3.2.2. Reliability and Measurement Error

The CIVIQ-20-Ar demonstrated excellent overall internal consistency, with a Cronbach’s alpha of 0.95 for the global index score, while the internal consistency of subscales ranged from 0.78 to 0.89. The Psychological Well-Being subscale had the highest internal consistency (α = 0.89), followed by the pain and physical function subscales (α = 0.88 and 0.87, respectively), while Cronbach’s alpha for the social functioning subscale was the lowest (α = 0.78), confirming that the instrument reliably measures the intended constructs (Table 2).

Test–retest reliability results for the Arabic CIVIQ-20 indicated no significant differences in global index scores between the two sessions (35.49 ± 18.12 and 36.08 ± 18.13, *p* > 0.05) and in each subscale (*p* > 0.05). In the test–retest subgroup (*n* = 60; 3–7-day interval), participants met the clinical stability threshold (GROC score −2 to +2). The CIVIQ-20-Ar showed very strong reproducibility with a global score ICC_(2,1)_ of 0.99 (95% CI: 0.98–0.99), while domain-specific ICC values ranged from 0.97 to 0.98.

The SEM and MDC for the Arabic CIVIQ-20 global index total score were 2.92 and 8.21, respectively. The SEM for each subscale ranged from 3.28 to 5.04, and the MDC ranged from 9.23 to 14.13. The resulting MDC95 values were lowest for the psychological (9.23 points) and pain (9.25 points) domains. Conversely, the social functioning and physical functioning domains exhibited slightly higher thresholds of 13.74 and 14.13 points, respectively.

#### 3.2.3. Validity

The I-CVI for the questionnaire ranged from 0.78 to 1.00: 16 of 20 questions scored 1.00; three items (Q3, Q17, and Q18) scored 0.89; and one item scored 0.78 (Q15). This indicates that the scale items were relevant and clear among the experts, while the S-CVI/Ave for the questionnaire was 0.97. No floor or ceiling effect was observed in the Arabic CIVIQ-20-Ar global index total score or in the four subscales.

Correlations between the global index and the four domains of CIVIQ-20-Ar and SF-36 are presented in Table 3.

Convergent validity of the CIVIQ-20-Ar was supported by significant negative correlations with the SF-36 domain and summary scores, consistent with all hypotheses. The global index scores of the CIVIQ-20-Ar had strong negative correlations with the SF-36′s physical component summary (r = −0.77, *p* < 0.001) and a moderate negative correlation with the mental component summary (r = −0.60, *p* < 0.001). Domain-specific analyses revealed moderate negative correlations in physical functioning (r = −0.54, *p* < 0.001), psychological functioning (r = −0.48, *p* < 0.001), and social functioning (r = −0.42, *p* < 0.001), and a strong negative correlation with pain (r = −0.79, *p* < 0.001).

Known-group validity was confirmed, as participants with more advanced CEAP stages (C5–6) reported significantly lower HRQoL (*p* < 0.01), with a consistent trend observed across all domains, including psychological (*p* < 0.001), physical functioning (*p* < 0.001), social functioning (*p* < 0.001), and the pain domain (*p* < 0.001) (Table 4). All seven construct validity hypotheses were accepted.

## 4. Discussion

Measuring health-related quality of life in participants with chronic venous insufficiency is essential for designing clinical treatment plans and tracking long-term rehabilitation outcomes. To our knowledge, this is the first study to translate and cross-culturally adapt the CIVIQ-20 questionnaire from English into Modern Standard Arabic, which is used across official settings and media throughout the Arab world. Our findings demonstrate that the CIVIQ-20-Ar is culturally acceptable, with strong internal consistency and excellent test–retest reliability. Regarding construct validity, the CIVIQ-20-Ar showed moderate-to-strong significant correlations with the SF-36, alongside robust discriminative validity.

The cross-cultural adaptation of the original English CIVIQ-20 into Modern Standard Arabic was rigorously performed in accordance with the guidelines established by Beaton et al. [22]. The translation process proved straightforward because of the instrument’s concise structure, ease of administration, and high feasibility. The questionnaire required only minor linguistic modifications. For instance, “going to parties” was adapted to “social events” (omitting specific references to “discos” and “cocktails”) to ensure cultural resonance, and frequency descriptors like “quite often” were refined for clinical and research clarity. Unlike other translation studies which required extensive adaptations to achieve cultural relevance [17], our modifications were minimal. This process produced a Modern Standard Arabic version (CIVIQ-20-Ar) that maintains semantic equivalence with the source instrument. Using Modern Standard Arabic ensures broad utility across all Arabic-speaking populations, avoiding the geographical limitations associated with regional dialects.

The CIVIQ-20-Ar demonstrated high overall internal consistency, with a global Cronbach’s alpha of 0.95 and domain-specific values ranging from 0.82 to 0.91. While an alpha coefficient above 0.80 is widely considered ideal for clinical outcome measures, values exceeding 0.90 must be interpreted with caution. As noted in the psychometric measurement literature, a global alpha of 0.95 may indicate certain item redundancy or conceptual overlap among closely linked items, particularly across the pain and physical functioning subscales. Nevertheless, this high internal consistency directly aligns with previously published international validations of the CIVIQ-20, including the original international validation study (α= 0.94) [16], Dutch (α = 0.92), Croatian (α = 0.94) [19], Turkish (α = 0.93) [20], Greek (α = 0.92) [21], Indonesian (α = 0.91) [39], and the original developmental version (α = 0.90) [14]. This suggests that while item redundancy may be inherent to the original 20-item structure, the Arabic version preserves the psychometric properties and inter-item reliability of the original instrument.

Internal consistency for the physical domain (α = 0.87) was comparable to the international validation study (α = 0.86) [16] and slightly higher than the French (α = 0.83) [14], Dutch (α = 0.82) [18], and Greek (α = 0.83) [21] versions. These results indicate that mobility items (e.g., stair climbing, kneeling, and brisk walking) have strong conceptual clarity and cultural relevance among Saudi patients, confirming the validity of assessing physical functional burden in Arabic clinical settings. The pain domain of the CIVIQ-20-Ar demonstrated excellent internal consistency reliability (α = 0.88) for evaluating both the frequency and severity of lower extremity discomfort. This internal consistency exceeded values reported in most previous studies, including the multi-country international study (α = 0.83) [16], the Greek validation (α = 0.82) [21], and the original French developmental study (α = 0.83) [16]. The high reliability of this subscale is clinically significant, as pain represents the predominant symptom motivating patients with chronic venous disease to seek medical intervention. Our findings confirm that the CIVIQ-20-Ar is a highly dependable instrument for assessing key health-related outcomes. Specifically, the psychological domain produced a Cronbach’s alpha of 0.89, reflecting robust internal consistency. This result aligns closely with both the multi-country international validation study (α = 0.89) [16] and the original French study (α = 0.90) [14], while exceeding the reliability reported in the Greek version (α = 0.77) [21]. The strong item homogeneity in this domain indicates that the Arabic version accurately captures the psychological burden of chronic venous disease. It effectively evaluates emotional responses such as feeling “on edge” or becoming “easily tired.” The Cronbach’s alpha coefficient of the social functioning subscale (α = 0.78) in the CIVIQ-20-Ar demonstrated higher reliability than the original French developmental version (α = 0.72) [14]; however, it was lower than the Greek adaptation (α = 0.83) [21]. Nevertheless, it is closely similar to the score obtained in the international validation study (α = 0.76) [16]. This improved consistency in the social domain relative to the original scale may be attributed to the cultural and religious importance of supportive social and familial support structures inherent in Saudi Arabian culture [40,41].

The test–retest reliability of the CIVIQ-20-Ar was evaluated using the ICC across a short 3–7-day interval. This timeframe was selected to minimize recall bias [14,21,30] while ensuring that underlying chronic venous disease severity remained clinically stable [14,42,43]. Our findings demonstrated excellent test–retest reliability, producing an ICC of 0.99 for the global index score. This result closely matches the original French developmental validation, which reported robust ICCs of 0.98 and 0.95 across two distinct cohorts using an identical time window [14]. Our findings also agree with the high reproducibility observed in the Greek validation (r = 0.98) over a 2-week interval [21]. This high level of reproducibility shows that the Arabic version is exceptionally stable when the patient’s health status remains unchanged. Conversely, lower reliability coefficients were reported in the Dutch (ICC = 0.86) [18] and Turkish (ICC = 0.80) [21] studies, which used a longer 4-week retest interval. Rather than reflecting instrument instability, this relative attenuation in reliability is likely due to natural clinical fluctuations in venous symptomatology over an extended timeframe. Notably, the large-scale international validation documented an ICC of 0.95 over a 2-week retest period [16].

Following COSMIN methodological recommendations [44], the CIVIQ-20-Ar was thoroughly evaluated for absolute reliability and minimal detectable changes. The global index score’s Standard Error of Measurement was 2.92 points. Although the original developmental study [14] did not report absolute reliability parameters, our SEM aligns closely with the 2.63 points reported in the Turkish validation [20], confirming acceptable measurement precision. The global index scale’s MDC was established at 8.21 points, representing the minimum score required to rule out measurement error at a 95% confidence level. Importantly, this value reflects statistical variation rather than clinical significance and should not be confused with a minimal clinically important difference (MCID). While our MDC is slightly higher than the 5.79 points observed in the Turkish cohort [20], this difference is likely due to variations in sample size and baseline heterogeneity. It provides an essential context for interpreting true score shifts. Crucially, the previous validation literature lacks domain-specific error indices; this study establishes the first baseline SEM and MDC parameters for the physical functioning, pain, psychological, and social functioning subscales. These domain-level benchmarks significantly enhance the precision of CIVIQ-20-Ar for tracking true longitudinal changes in Arabic-speaking healthcare settings.

In this study, the CIVIQ-20-Ar demonstrated excellent content validity across relevance, clarity, and cultural appropriateness. The expert panel assessment yielded item-level content validity indices (I-CVI) ranging from 0.78 to 1.00, a scale-level average CVI (S-CVI/Ave) of 0.97, and a universal agreement score (S-CVI/UA) of 0.80. These values comfortably exceed established psychometric thresholds, reflecting strong expert consensus regarding item relevance [35,36]. Pilot testing further confirmed that participants found the instrument clear, meaningful, and easy to complete. Additionally, floor (3.6–7.9%) and ceiling (0.7–1.4%) effects across all domains were minimal and well below the widely accepted 15% psychometric threshold. These results closely mirror the floor (0.9–8.5%) and ceiling (0.0–0.3%) ranges reported in the large-scale international validation study [16], confirming that the CIVIQ-20-Ar can capture the full spectrum of disease severity without risk of measurement saturation.

Our findings demonstrate robust convergent validity, confirming the hypothesized correlations between the CIVIQ-20-Ar global index, its subscales, and the SF-36. Specifically, the CIVIQ-20-Ar global index correlated more strongly with the SF-36′s physical component summary (r = −0.77, *p* < 0.001) while maintaining a moderate correlation with the mental component summary (r = −0.60, *p* < 0.001). These results closely parallel the findings of the Dutch validation study by Biemans et al. [18]. Consistent with the international literature, the CIVIQ-20-Ar correlated more strongly with physical summary scores than mental summary scores, confirming that the instrument predominantly captures physical impairment and pain-related functional limitations rather than psychological status alone [18,45]. Furthermore, the CIVIQ-20-Ar’s subscales demonstrated moderate-to-strong convergent validity with corresponding SF-36 domains. The strongest association was observed within the pain domain (r = −0.79, *p* < 0.001), which aligns closely with the Greek validation reported by Erevnidou et al. [21]. This consistency highlights that localized bodily symptoms are the primary manifestation of chronic venous disease in various cultural groups. Crucially, the CIVIQ-20-Ar consistently maintained the expected directional relationships across all domains, with all correlation coefficients exceeding the predefined threshold (r = −0.40).

Another key dimension supporting the theoretical construct of the CIVIQ-20-Ar is its ability to differentiate effectively between patient groups based on clinical severity. Our findings demonstrate that both the subscale scores and the overall global index of the CIVIQ-20-Ar can successfully distinguish between patients in early clinical stages of chronic venous disease and those with more advanced disease severity according to the CEAP classification (e.g., distinguishing early stages C1–2 from advanced stages, C3–4, and C5–6; *p* < 0.01). This known-group validity confirms that the Arabic version reflects the progressive deterioration of HRQoL associated with advancing venous pathology. This aligns with the international validation of the Dutch [18] and Cortina [19] versions, reinforcing the CIVIQ-20-Ar as a clinical tool capable of reflecting disease burden across the full clinical spectrum of chronic venous insufficiency.

In the present study, all seven hypotheses were validated, confirming the CIVIQ-20-Ar’s robust psychometric soundness. It provides strong empirical evidence for both its convergent validity and known-group validity. Consequently, the CIVIQ-20-Ar can be considered a valid, reliable, and culturally adapted instrument for assessing health-related quality of life (HRQoL) in Arabic-speaking patients across the spectrum of chronic venous disease. The CIVIQ-20-Ar demonstrates notable methodological strengths, including a rigorous cross-cultural adaptation process and the application of standardized psychometric protocols that ensure conceptual and semantic equivalence. As the first formal adaptation of this instrument into Arabic for clinical and research applications, this study establishes a strong psychometric foundation supported by high internal consistency, test–retest reliability, and a defined minimal detectable change.

This study has several limitations that should be acknowledged. First, convenience sampling of patients recruited from two clinical centers in Riyadh may limit the generalizability of our findings, particularly from patients managed in primary care settings or presenting with symptoms. Second, although rigorous statistical analyses confirmed convergent validity and known-group validity, the internal structural validity of the CIVIQ-20-Ar was not formally evaluated using factor analysis. Exploratory Factor Analysis and Confirmatory Factor Analysis were not performed to test whether the original four-domain structure (pain, physical, psychological, and social) remains invariant in the Arabic population. While internal consistency was strong across domains, the large overall scale homogeneity (alpha = 0.95) may also suggest item redundancy. Future prospective studies with larger samples should perform structural factor modeling to verify the underlying dimensional structure of the Arabic version. Finally, because this study employed a cross-sectional design, the longitudinal responsiveness of the CIVIQ-20-Ar was not assessed. Future prospective, multi-center studies with larger sample sizes are recommended to perform factorial structure analysis and evaluate the longitudinal responsiveness and minimal clinically important difference in the Arabic version.

## 5. Conclusions

The CIVIQ-20-Ar questionnaire showed satisfactory content validity, convergent construct validity, known-group validity, excellent test–retest reliability, and substantial internal consistency. Therefore, vascular surgeons, phlebologists, and rehabilitation specialists can use the CIVIQ-20-Ar in daily clinical practice and research to evaluate health-related quality of life in the Arabic-speaking population with chronic venous disease.

## Figures and Tables

**Table 1:** Demographic and clinical characteristics of the participants (*N* = 139).

	*n* (%) or Mean ± SD
Age (yrs.)	47.13 ± 11.93
BMI (kg/m^2^)	29.13 ± 6.28
Gender	
Male	22 (15.83%)
Female	117 (84.17%)
Marital status	
Married	120 (86.33%)
Not married	19 (13.67%)
Education level	
High school or less	43 (30.94%)
Bachelors	81 (58.27%)
Postgraduate degree	15 (10.79%)
Work status	
Employee	71 (51.08%)
Not working/retired	68 (48.92%)
Smoking Status	
Smoker	9 (6.47%)
Non-smoker	130 (93.53%)
CEAP Clinical Stage	
C1–C2	89 (64.00%)
C3–C4	31 (22.00%)
C5–C6	19 (14.00%)
CIVIQ-20-Ar Domain Scores	
Physical functioning	37.94 ± 24.96
Pain	35.56 ± 23.8
Social functioning	36.51 ± 22.3
Psychological well-being	25.43 ± 19.45
Global index total score	31.67 ± 19.95
SF-36 summary scores	
Mental component summary	64.73 ± 20.26
Physical component summary	62.98 ± 25.29

All data are represented as absolute frequencies and percentages (*n*, %) unless otherwise specified. BMI = Body Mass Index; CEAP = Clinical, Etiological, Anatomical, and Pathophysiological Classification; CIVIQ-20-Ar = the Arabic version of Chronic Venous Insufficiency Questionnaire-20; PCS = Physical Component Summary; SD = Standard Deviation; SF-36 = 36-Item Short Form Health Survey.

**Table 2:** Internal consistency reliability, test–retest reliability, SEM, and MDC (95% MDC) for CIVIQ-20-Ar.

CIVIQ-20-Ar	Internal Consistency α	Test–Retest Reliability			SEM	MDC_95_
		Time 1 Mean ± SD	Time 2 Mean ± SD	ICC (95% CI)		
Pain	0.88	43.75 ± 23.99	42.42 ± 23.27	0.97 (0.95, 0.98)	3.29	9.25
Physical Functioning	0.87	28.07 ± 17.85	29.97 ± 18.17	0.97 (0.96, 0.98)	5.04	14.13
Social Functioning	0.78	41.92 ± 20.51	41.41 ± 20.51	0.97 (0.95, 0.98)	4.89	13.74
Psychological Well-being	0.89	39.11 ± 22.26	39.49 ± 22.28	0.98 (0.96, 0.99)	3.28	9.23
Global Index Total Score	0.95	35.49 ± 18.12	36.08 ± 18.13	0.99 (0.98, 0.99)	2.92	8.21

α = Cronbach’s alpha; CI = Confidence Interval; CIVIQ-20-Ar = Chronic Venous Insufficiency Questionnaire (Arabic version); ICC = Intraclass Correlation Coefficient (two-way random effects and absolute agreement); MDC_95_ = Minimal Detectable Change at the 95% Confidence Level; SEM = Standard Error of Measurement; SD = Standard Deviation.

**Table 3:** Hypotheses and correlation coefficients for convergent (*n* = 6) and known-group validity (*n* = 1).

	Convergent Validity Hypothesis (1–6)	Hypothesized Correlation	Calculated Pearson’s Correlation Coefficient (r)	Hypothesis Confirmed?
1	The physical domain of CIVIQ-20-Ar will have a moderate correlation with the SF-36′s physical functioning subscale.	Negative (r ≥ −0.40)	−0.54 **	Yes
2	The psychological domain of CIVIQ-20-Ar will have a negative moderate correlation with the SF-36′s emotional role limitation subscale.	Negative (r ≥ −0.40)	−0.48 **	Yes
3	The social domain of CIVIQ-20-Ar will have a negative moderate correlation with the SF-36′s social functioning subscale.	Negative (r ≥ −0.40)	−0.42 **	Yes
4	The pain domain of CIVIQ-20-Ar will have a negative moderate correlation with the SF-36′s bodily pain subscale.	Negative (r ≥ −0.40)	−0.79 **	Yes
5	The total score of CIVIQ-20-Ar will have a negative moderate correlation with the SF-36′s physical component summary.	Negative (r ≥ −0.40)	−0.77 **	Yes
6	The total score of CIVIQ-20-Ar will have a negative moderate correlation with the SF-36′s mental component summary.	Negative (r ≥ −0.40)	−0.60 **	Yes
	Known-group validity hypotheses			
7	Participants with more advanced stages of CVD will report significantly lower HRQoL compared to those in earlier stages.	*p* < 0.05	*p* < 0.01	Yes

CIVIQ-20-Ar = Chronic Venous Insufficiency Questionnaire (Arabic version); SF-36 = 36-Item Short Form Health Survey. ** *p* < 0.001.

**Table 4:** Known-group construct validity: CIVIQ-20-Ar score differences across CEAP clinical stages.

Arabic CIVIQ-20	CEAP C1–C2 89 (64%)	CEAP C3–C4 31 (22%)	CEAP C5–C6 19 (14%)	*p*
	Median (IQR)	Median (IQR)	Median (IQR)	
GIS	18.75(10.00–27.50) ^†^	45.25 (38.75–51.25)	63.75(58.75–73.75)	<0.001
Psychological well-being	13.89 (5.56–20.83) ^†^	36.11 (30.56–41.67)	61.11 (50–69.45)	<0.001
Physical functioning	25 (9.37–34.38) ^†^	56.25 (43.75–68.75)	68.75 (62–81.25)	<0.001
Social functioning	25 (16.67–33.34) ^†^	58.33 (33.33–58.33)	66.67 (50–75)	<0.001
Pain domain	18.75 (6.25–37.50) ^†^	56.25 (43.75–56.25)	68.75 (56.25–81.25)	<0.001

Values are presented as median (interquartile range). CEAP: Clinical–Etiologic–Anatomic–Pathophysiologic Classification; CIVIQ-20: Chronic Venous Insufficiency Quality of Life Questionnaire (20-item version); GIS: Global Index Score; IQR: Interquartile Range. ^†^ Statistically significant differences were observed across CEAP stages (*p* < 0.001) using the Kruskal–Wallis test, with post hoc pairwise comparisons indicating significantly lower QoL scores in early CEAP stages.

## Data Availability

The data supporting this study’s findings are available from the corresponding author upon reasonable request.

## Supplementary Materials

The following supporting information can be downloaded at: https://www.mdpi.com/article/10.3390/jcm15197558/s1.

## Abbreviations

The following abbreviations are used in this manuscript:

BMI	Body Mass Index
CEAP	Clinical, Etiological, Anatomical, and Pathophysiological Classification
CI/CIs	Confidence Interval/Confidence Intervals
CIVIQ-20	20-item Chronic Venous Insufficiency Quality-of-Life Questionnaire
CIVIQ-20-Ar	Chronic Venous Insufficiency Questionnaire (Arabic version)
COSMIN	Consensus-based Standards for the Selection of Health Measurement Instruments
CVI	Chronic Venous Insufficiency
EFA	Exploratory Factor Analysis
HRQoL	Health-Related Quality of Life
I-CVI	Item-level Content Validity Index
ICC_(2,1)_	Intraclass Correlation Coefficient (specifically, two-way random intraclass correlation coefficient)
KMO	Kaiser–Meyer–Olkin Measure of Sampling Adequacy
MCS	Mental Component Summary
MDC_95_	Minimal Detectable Change at a 95% Confidence Level
NHP	Nottingham Health Profile
PCS	Physical Component Summary
PROMs	Patient-Reported Outcome Measures
S-CVI	Scale-level Content Validity Index
S-CVI/Ave	Scale-level Content Validity Index Average Method
S-CVI/UA	Scale-level Content Validity Index Universal Agreement Method
SD	Standard Deviation
SEM	Standard Error of Measurement
SF-36	36-item Short Form Health Survey
SIP	Sickness Impact Profile
SPSS	Statistical Package for the Social Sciences
