# Supplementary material for: Cross-Cultural Adaptation and Psychometric Validation of the Arabic Chronic Venous Insufficiency Quality-of-Life Questionnaire (CIVIQ-20) in Saudi Arabia
Source: J Clin Med. 2026 Sep 29;15(19):7558. doi: 10.3390/jcm15197558 (PMC13665721; doi:10.3390/jcm15197558)
Supplement: Supplementary file 1 [file jcm-15-07558-s001.zip › jcm-4545017-supplementary.pdf]

استبيان جودة الحياة للقصور الوريدى المزمن

١) خلال الأسابيع الأربعة الماضية، في حال أنك شعرت بألم في مفصل الكاحل أو الساق، ما مدى شدة الألم
(ضع دائرة على الرقم الذي يمثل الإجابة الصحيحة)

لا يوجد ألم - ألم خفيف - ألم متوسط - ألم شديد - ألم شديد للغاية

١ ٢ ٣ ٤ ٥

٢) إلى أي مدى خلال الأسابيع الأربعة الماضية شعرت بإزعاج أو تقييد أثناء عملك أو أنشطتك اليومية؛ بسبب متاعب الساق؟ (ضع دائرة على الرقم الذي يمثل الإجابة الصحيحة)

لم أشعر بذلك - بشكل طفيف - بشكل متوسط - بشده - بشدة للغاية

١ ٢ ٣ ٤ ٥

٣)هل كان نومك سيئاً خلال الأسابيع الأربعة الماضية بسبب مشاكل ساقيك؛ وكم مرة يحدث ذلك؟
(ضع دائرة على الرقم الذي يمثل الإجابة الصحيحة)

أبداً - نادراً - غالباً - كثيرا ً - كل ليلة

١ ٢ ٣ ٤ ٥

- خلال الأسابيع الأربعة الماضية، إلى أي مدى آلام الساق أزعجت/ قيدت نشاطك الحركي الموضح أدناه؟

(حدد مدى انزعاجك أو تقييدك بوضع دائرة على الرقم الذي يمثل الإجابة الصحيحة في كل جملة أدرجت في العمود الذي على اليمين في القائمة الآتية :)

**نوع الحركة أو النشاط**	**مدى الإزعاج أو التقييد عند القيام بها**				
	**دون إزعاج أو تقييد**	**إزعاج وتقييد بسيط**	**إزعاج وتقييد متوسط**	**إزعاج وتقييد شديد**	**يستحيل القيام بها**
٤- الوقوف لوقت طويل	١	٢	٣	٤	٥
٥- صعود الدرج	١	٢	٣	٤	٥

٦- الجلوس متربعاً أو الركوع	١	٢	٣	٤	٥
٧- المشي السريع	١	٢	٣	٤	٥
٨- السفر بالسيارة أو الحافلة أو الطائرة	١	٢	٣	٤	٥
٩- الأعمال المنزلية مثل العمل في المطبخ، حمل الأطفال، الكوي، تنظيف الأرضية والأثاث، الأعمال اليدوية	١	٢	٣	٤	٥
١٠- الذهاب لحفلات الزفاف أو المناسبات الاجتماعية	١	٢	٣	٤	٥
١١- الأنشطة الرياضية، بذل مجهود بدني شاق	١	٢	٣	٤	٥

- متاعب الساقين يمكن أن يكون لها تأثير على السلوك؛ حدد إلى أي مدى تنطبق الجمل الآتية على الحالة التي مررت بها خلال الأسابيع الأربعة الماضية؟ (ضع دائرة على الرقم الذي تعتقد أنه الجواب الصحيح أمام كل جملة أدرجت في العمود الذي على اليمين)

**الجملة**	**الإجابة**				
	**أبداً**	**بشكل طفيف**	**بشكل متوسط**	**بشده**	**بشدة بالغة**
١٢- أشعر بالضيق	١	٢	٣	٤	٥
١٣- كنت أتعب بسرعه	١	٢	٣	٤	٥
١٤- شعرت بأني عبء على الأخرين	١	٢	٣	٤	٥
١٥- يجب أن أتخذ دائمًا الاحتياطات (مثل تمديد ساقي لتجنب الوقوف لفترة طويلة ...)	١	٢	٣	٤	٥
١٦- أشعر بالحرج من إظهار ساقي	١	٢	٣	٤	٥
١٧- أغضب بسرعة	١	٢	٣	٤	٥
١٨- أشعر بالإعاقة	١	٢	٣	٤	٥
١٩- أجد صعوبة عند الخروج من البيت صباحاً	١	٢	٣	٤	٥
٢٠- لم يكن لدي الرغبة في الخروج من البيت	١	٢	٣	٤	٥
